# Does Mindfulness Enhance Critical Thinking? Evidence for the Mediating Effects of Executive Functioning in the Relationship between Mindfulness and Critical Thinking

**DOI:** 10.3389/fpsyg.2015.02043

**Published:** 2016-01-19

**Authors:** Chris Noone, Brendan Bunting, Michael J. Hogan

**Affiliations:** ^1^School of Psychology, National University of IrelandGalway, Ireland; ^2^School of Psychology, Ulster UniversityColeraine, UK

**Keywords:** self-regulation, higher-order cognition, dispositional mindfulness, dual-process theory, critical thinking

## Abstract

Mindfulness originated in the Buddhist tradition as a way of cultivating clarity of thought. Despite the fact that this behavior is best captured using critical thinking (CT) assessments, no studies have examined the effects of mindfulness on CT or the mechanisms underlying any such possible relationship. Even so, mindfulness has been suggested as being beneficial for CT in higher education. CT is recognized as an important higher-order cognitive process which involves the ability to analyze and evaluate evidence and arguments. Such non-automatic, reflective responses generally require the engagement of executive functioning (EF) which includes updating, inhibition, and shifting of representations in working memory. Based on research showing that mindfulness enhances aspects of EF and certain higher-order cognitive processes, we hypothesized that individuals higher in facets of dispositional mindfulness would demonstrate greater CT performance, and that this relationship would be mediated by EF. Cross-sectional assessment of these constructs in a sample of 178 university students was achieved using the observing and non-reactivity sub-scales of the Five Factor Mindfulness Questionnaire, a battery of EF tasks and the Halpern Critical Thinking Assessment. Our hypotheses were tested by constructing a multiple meditation model which was analyzed using Structural Equation Modeling. Evidence was found for inhibition mediating the relationships between both observing and non-reactivity and CT in different ways. Indirect-only (or full) mediation was demonstrated for the relationship between observing, inhibition, and CT. Competitive mediation was demonstrated for the relationship between non-reactivity, inhibition, and CT. This suggests additional mediators of the relationship between non-reactivity and CT which are not accounted for in this model and have a negative effect on CT in addition to the positive effect mediated by inhibition. These findings are discussed in the context of the Default Interventionist Dual Process Theory of Higher-order Cognition and previous studies on mindfulness, self-regulation, EF, and higher-order cognition. In summary, dispositional mindfulness appears to facilitate CT performance and this effect is mediated by the inhibition component of EF. However, this relationship is not straightforward which suggests many possibilities for future research.

## Introduction

Despite its origins as a way of cultivating clarity of thought, among the collection of studies on mindfulness conducted in recent years, few have been concerned with the link between mindfulness and thinking skills in typically developing individuals. Assessments that attempt to capture the thinking skills of people in real-world situations can be found in the body of literature focused on critical thinking (CT; Ku, 2009; Butler, 2012). CT is recognized as an important higher-order cognitive process which involves the ability to analyze and evaluate evidence and arguments without bias from experience and prior knowledge. The application of CT requires a non-automatic response to a problem situation in order to avoid heuristic and biased thinking (West et al., 2008). Such non-automatic, critical, and reflective responses generally require the engagement of executive functioning (EF) – monitoring, updating, and switching between representations in working memory – and are regarded as involving deliberative processes, generally referred to as Type 2 processes by Dual-Processing theorists (Evans and Stanovich, 2013). Mindfulness is often described as a process consisting of two components: present-moment attentional focus coupled with non-reactive monitoring of one’s ongoing experience (Bishop et al., 2004). While an emerging body of theoretical and empirical work has linked mindfulness with enhanced EF (Chambers et al., 2007; Josefsson and Broberg, 2011; Tang et al., 2012; Teper and Inzlicht, 2013), little empirical work has been carried out on the relationship between EF and CT (Sanz de Acedo Lizarraga et al., 2012). Furthermore, though mindfulness has been shown to facilitate certain types of higher-order cognition, including insight problem-solving (Ostafin and Kassman, 2012; Wen et al., 2013), moral reasoning and ethical decision-making (Cottone and Javier, 2007; Ruedy and Schweitzer, 2011; Shapiro et al., 2012), the relationship between mindfulness and CT has not been investigated. In light of these identified gaps in the literature, the current study sought to investigate the relationship between dispositional mindfulness, EF, and CT skills.

Though there is overwhelming consensus that CT skills should be cultivated in educational and occupational settings, attempts to converge on an operational definition of CT have been far from unanimous (Dwyer et al., 2014). Common to most conceptualisations of CT is the ability to evaluate arguments and evidence without influence from one’s own prior belief and experience (West et al., 2008). One influential definition proposes that CT involves the application of particular cognitive skills including analysis, evaluation, and inference skills, in a purposeful, reasoned, and goal-directed manner (Halpern, 1998; Facione, 2013). It has been demonstrated that the appropriate execution of these CT skills is influenced by the presence of specific dispositions toward thinking, including a disposition toward truth-seeking, open mindedness, prudence, diligence, and flexibility (Facione, 2013; Dwyer et al., 2014). CT performance also depends on a person’s awareness that a particular thinking skill is required, that the ongoing execution of the skill is adequate, and the ability to monitor and exert control over thinking processes (Halpern, 1998). Together, these have been referred to as the self-regulatory functions of metacognition (Halpern, 1998; Ku and Ho, 2010; Dwyer et al., 2014). Studies focusing on both the direct assessment of CT and the avoidance of heuristic and biased thinking have shown that the ability to monitor and control ongoing thought processes results in better real-world outcomes (Butler, 2012; Butler et al., 2012), particularly when making decisions in personal, professional, and legislative domains (Kahneman and Tversky, 2000; Hastie and Dawes, 2001; Myers, 2002; Hilton, 2003; Sunstein, 2005; Baron et al., 2006; Lichtenstein and Slovic, 2006; Reyna and Farley, 2006; Reyna and Lloyd, 2006). Few studies have examined the real-world cognitive outcomes of mindfulness though one study did show that dispositional mindfulness was related to fewer experiences of cognitive failures in daily life (Herndon, 2008).

This study examines a possible link between dispositional mindfulness and CT. However, there are at least two competing views as to how mindfulness might relate to CT. One view suggests that mindfulness is either not related to CT or even a hindrance to effective CT due to its association with acceptance and non-elaborative, or non-reactive processing (Brendel, 2015). Another view suggests that mindfulness facilitates effective CT due to its association with improved self-regulation (Shapiro et al., 2011). In light of an emerging body of research demonstrating a positive relationship between mindfulness, self-regulation, and EF we propose that the latter view may be true. However, a link between mindfulness and CT has not been established in the literature and this may depend on a range of factors including the form of mindfulness investigated, the particular CT skill assessed, and the characteristics of study participants. The current study focused on younger adults and examined the relationship between dispositional mindfulness as measured using a short form of the Five Factor mindfulness Questionnaire (Bohlmeijer et al., 2011) and CT performance assessed using the Halpern Critical Thinking Assessment (Halpern, 2010). Though the relationship between dispositional mindfulness and CT has not been directly assessed yet, a positive relationship between dispositional mindfulness and related constructs such as ethical decision-making, insight problem-solving and creative thinking has been demonstrated (Ruedy and Schweitzer, 2011; Ostafin and Kassman, 2012; Baas et al., 2014). Dispositional mindfulness has also been found to be negatively correlated with engagement in intuitive thinking (Remmers et al., 2014). In light of recent research suggesting a link between mindfulness, self-regulation, and EF (Feltman et al., 2009; Tang and Posner, 2009; Sahdra et al., 2011; Moore et al., 2012; Lyvers et al., 2013; Morgan et al., 2013; Ostafin et al., 2013a) and work suggesting a link between self-regulation, EF, and CT performance (West et al., 2008; Sanz de Acedo Lizarraga et al., 2012; Evans and Stanovich, 2013; Dwyer et al., 2014), the current study sought to investigate whether the relationship between dispositional mindfulness and CT performance is mediated by EF performance.

The link between mindfulness and self-regulatory processes can best be explored by considering the currently most highly endorsed operational definition of mindfulness which conceptualizes mindfulness as consisting of two components which pertain to the focus and quality of attention, respectively, i.e., present-moment attentional focus coupled with non-reactive monitoring of one’s ongoing experience (Bishop et al., 2004). This study focuses on dispositional mindfulness, a construct which reflects the tendency to engage in this non-reactive, present-moment attention (Brown and Ryan, 2003). Early teachings on mindfulness suggested that a dispositional tendency to engage in mindful attention is an innate trait as well as something which can be trained (Rau and Williams, 2015). Dispositional mindfulness is considered to be stable over time but can be modified through certain meditative practices, for example, practices involving Focused Attention and Open Monitoring (Davidson and Kaszniak, 2015; Kiken et al., 2015), integrative mind-body practices such as yoga, tai chi and qigong (Schure et al., 2008; Caldwell et al., 2010) and particular exercises developed within the traditions of Dialectical Behavior Therapy (Perroud et al., 2012) and Acceptance and Commitment Therapy (Ciarrochi et al., 2010). Recent research has shown that over the course of a mindfulness-based meditation training program individual trajectories of change in the ability to engage a mindful state is associated with both increases in dispositional mindfulness and psychological health (Kiken et al., 2015). This adds to research demonstrating associations between dispositional mindfulness in non-meditators and adaptive outcomes such as increased life satisfaction (Kong et al., 2014), better emotional regulation (Goodall et al., 2012), lower depression and anxiety symptoms (Desrosiers et al., 2013), less negative and more positive affect (Short et al., 2015), and increased self-esteem (Rasmussen and Pidgeon, 2011). It is also consistent with studies on mindfulness-based interventions that demonstrated increases in dispositional mindfulness which correlated with psychological health benefits (Kiken et al., 2015). A range of measures have been developed to assess dispositional mindfulness and across these the two-factor structure of mindfulness tends to be supported (Rau and Williams, 2015).

The first component of mindfulness involves present-moment attentional focus. When attention is focused on the present moment, internal and external stimuli are observed and brought into awareness. This allows affective cues which are normally overlooked to be noticed. It has been suggested that one function of such affective cues is to indicate that the current state an individual is in is inconsistent with their goal state and therefore control needs to be exerted – a process sometimes referred to as conflict monitoring (Teper et al., 2013). Enhanced present-moment awareness and conflict monitoring has been associated with greater sensitivity to perceptual cues (Anicha et al., 2011; Teper and Inzlicht, 2013; Teper et al., 2013) and enhanced executive control skills, including the ability to update and switch between thought-action representations in working memory (Bishop et al., 2004; Moore and Malinowski, 2009).

Non-reactivity – the second component of mindfulness – involves executive control to inhibit elaboration and/or suppression of affective cues. This allows for the early engagement of emotion regulation before intense emotional reactivity to the attended thoughts, feelings, and sensations can occur (Teper et al., 2013). Notably, research suggests that the two component skills of present-moment awareness and non-reactivity follow distinct developmental trajectories which may vary across individuals (Lilja et al., 2012). These developmental trajectories can be tracked using validated self-report questionnaires which assess the components of dispositional mindfulness such as the extensively used Five Factor Mindfulness Questionnaire employed in this study (Sauer et al., 2013). These questionnaires can also reveal the extent to which those without mindfulness training tend to engage mindful attention (Brown and Ryan, 2003).

As such, the predominant two-component operationalisation of mindfulness implies both monitoring and control, skills which are inherently self-regulatory (Bishop et al., 2004). Importantly, evidence for improved self-regulation of behavior as a result of mindfulness comes from studies on healthy eating (Jordan et al., 2014), procrastination (Sirois and Tosti, 2012), smoking cessation (Libby et al., 2012), persistence (Evans et al., 2009), and alcohol intake (Ostafin et al., 2012). Furthermore, evidence for improved self-regulation of emotions and thoughts comes from the extensive literature on the clinical benefits of mindfulness (Dowd et al., 2015; See reviews by Baer, 2003; Hofmann et al., 2010; Veehof et al., 2011), which has reported reductions in anxiety, depression, pain interference, and catastrophic thinking as a result of mindfulness training.

In cognitive models of self-regulation, the mobilization of self-regulatory resources is characterized by the effective operation of the EFs that support and govern working memory. Although many models of EF have been developed, there is an emerging consensus that EF involves three basic processes: updating, inhibition, and shifting. Updating refers to the active revision and monitoring of thinking; shifting refers to switching between tasks or mental sets; and inhibition refers to the active, deliberate suppression of thoughts or responses and the maintenance of attention on goal-relevant information (Miyake et al., 2000; Miyake and Friedman, 2012). The updating and maintenance of working memory is vital for the accurate active representation of goals and goal-related information (Hofmann et al., 2012). Furthermore, greater working memory capacity protects goal representations from thought intrusions and decreases mind-wandering. This is often referred to as goal shielding (Brewin and Smart, 2005). Greater inhibitory control has been linked to more successful goal shielding and self-regulation in behaviors ranging from eating behavior to sexual fidelity (Hofmann et al., 2008; Nederkoorn et al., 2010; Pronk et al., 2011). More generally, EFs support the coordination of thoughts and actions in a goal-directed manner and are essential for success in education, work, and everyday living (Hofmann et al., 2012).

Research also suggest that the EF processes of updating, inhibition, and shifting support higher-order processes involved in problem-solving (Burton et al., 2006), metacognition (Fernandez-Duque et al., 2000), and decision-making (Del Missier et al., 2010). EFs are particularly important in situations or tasks which are novel or where learned automatic responses are not adaptive and therefore additional control is required (Hofmann et al., 2012). Situations requiring CT fulfill these criteria and EFs may be important to sustain ongoing efforts at CT. Furthermore, mindfulness may support EFs (Chiesa et al., 2011). Considering the operational definition of mindfulness, which involves present-moment awareness and non-reactivity, we propose a relationship between components of mindfulness and component EFs. The sustained attention to present-moment experience developed through mindfulness practice likely requires the ability to switch attention between stimuli in current experience and back to current experience when the mind wanders (Bishop et al., 2004). In the language of working memory theory, this implies continuous updating of the thought-action representations which make up the contents of working memory as current experience changes and shifting between these thought-action representations (Teper and Inzlicht, 2013). Engaging this present-moment attention in a non-reactive manner then requires the inhibition of elaborative processing of such representations (Hayes and Shenk, 2004; Holas and Jankowski, 2012). Recent research has shown that EF mediates the relationship between mindfulness and positive and negative affect (Short et al., 2015) but no studies have examined the indirect effects of specific mindfulness facets through specific components of EF on outcomes usually associated with CT ability.

A number of studies have reported direct effects of mindfulness on specific components of EF. For example, several studies show a positive effect of mindfulness practice on the ability to inhibit automatic responses. Mindfulness practice over a 6 weeks period improved performance on a backward inhibition task (Greenberg et al., 2012a) and eight sessions of mindfulness-based cognitive therapy improved performance on the Hayling task (Heeren et al., 2009) – measures which are commonly used to assess inhibition. Studies employing the Stroop task have found better performance in experienced meditators than novices (Chan and Woollacott, 2007), after a 15 min mindfulness meditation (Wenk-Sormaz, 2005) and following a 6-weeks mindfulness training (Allen et al., 2012). However, one study reported no improvement in Stroop performance after an 8-weeks mindfulness-based stress reduction program (MBSR; Anderson et al., 2007).

Studies have also reported a positive link between mindfulness and the ability to switch between thought-action representations in working memory. Chambers et al. (2007) examined the effects of self-reported increases in mindfulness following a 10-days mindfulness retreat and found that improvement in switching performance was significantly correlated with increases in the tendency to employ mindful attention. Relative to a control condition, this experimental group showed improvement on the Internal Switching task, which measures switching between positive and negative affective words (Chambers et al., 2007). Using the Attention Network Task, Jha et al. (2007) found that relative to controls, participants in a MBSR course showed improved attention switching ability. In another study by Hodgins and Adair (2010), participants in a mindfulness training group performed better than controls on a task requiring participants to switch between visual perspectives. However, null effects have been reported in other studies. For example, Lykins et al. (2010) found no differences between novice and experienced meditators on the Color Trails test, and Anderson et al. (2007) found no change in performance on an attention switching task after an 8-weeks MBSR course.

Though no studies have examined the effects of mindfulness practice on working memory updating specifically, the effects of mindfulness on working memory capacity has been examined. One study reported that, after a 10-days mindfulness retreat, working memory capacity improved in a mindfulness group compared to a waitlist control-group (Chambers et al., 2007). Also, in a study that compared mindfulness training with an active control-group, working memory capacity showed improvement in the mindfulness group indexed by both the forward/backward digit span of the WAIS and the n-back task (Zeidan et al., 2010). Furthermore, in a military group prior to deployment, the deleterious effects of stress on working memory capacity were buffered against for those participants who reported high levels of mindfulness practice during an intervention (Jha et al., 2010).

Having presented evidence for the claim that mindfulness broadly supports self-regulation and that a key mechanism underlying this effect may be the enhancement of EF, the link between self-regulatory processes, EFs, and CT will now be discussed. In doing so, it is useful to consider dual processing accounts of higher-order cognition as a useful theoretical framework which can integrate the findings discussed in previous sections. In these accounts, researchers have proposed that two separate cognitive systems are available to us when higher-order cognition such as judgment, reasoning, decision-making or problem-solving is to be carried out (Evans, 2003, 2011; Kahneman, 2011; Evans and Stanovich, 2013). Indeed, Hart et al. (2013) suggested Kahneman’s (2011) description of dual-process theory as a framework for explaining the cognitive benefits of mindfulness interventions. They suggested that mindfulness prompts the self-regulation of attention which activates system 2 or reflective thinking. However, the Default-interventionist dual process theory of higher-order cognition may offer a more refined view as it specifies the mechanisms by which this self-regulation occurs and the conditions under which it will lead to an optimal response (Evans and Stanovich, 2013). This particular theory suggests that higher-order cognition, such as effective CT, requires inhibition of default Type 1 processes, which by definition exert minimal working memory load and occur automatically in response to stimuli (West et al., 2008). It is assumed that CT skills involve working memory driven Type 2 processes, which are typically slow, limited in capacity, conscious, and controlled. These Type 2 processes will be most effective when working memory is being updated efficiently as new information arises. However, exactly how EF relates to CT has not been investigated empirically (Sanz de Acedo Lizarraga et al., 2012). As noted above, mindfulness facilitates the executive mechanisms of working memory which are critical to engaging Type 2 processes. Furthermore, mindfulness training does appear to be beneficial for other higher-order thinking skills which may also depend on EF such as insight problem-solving (Ostafin and Kassman, 2012; Wen et al., 2013), moral reasoning and ethical decision-making (Cottone and Javier, 2007; Ruedy and Schweitzer, 2011; Shapiro et al., 2012). Each of these studies emphasized the non-automatic orientation to experience that mindfulness brings and which is characteristic of the engagement of EF and Type-2 processing but they did not examine whether EF mediated the effect of mindfulness on these cognitive outcomes. Therefore, we hypothesize that mindfulness may facilitate CT skills, and engagement of Type 2 processing in general, via enhancement of the EFs governing working memory. We explore this possibility by testing whether EF mediates the relationship between mindfulness and CT.

In light of the available evidence, several more specific predictions can be made about how components of mindfulness, EF, and CT are related. The current study adopted a multiple indicator individual differences study approach to test the following hypotheses using structural equation modeling (SEM):

1. First, it is hypothesized that the measurement model for mindfulness will support the two-component conceptualisation of mindfulness with observing representing present-moment awareness and non-reactivity representing non-reactive monitoring of one’s ongoing experience.
2. Second, it is hypothesized that the measurement model for EF will support the three-component unity/diversity model which proposes that updating, inhibition, and shifting are related but distinct components of EF.
3. Third, it is hypothesized that present-moment awareness and non-reactivity will be positively associated with CT performance. Notably, present-moment mindful awareness, often referred to as observing, has been associated with an enhanced ability to pick up relevant information (Carson and Langer, 2006), whereas non-reactivity has been shown to interrupt the biasing of information processing by both emotional state (Kiken and Shook, 2011) and temperament (Feltman et al., 2009). Using appropriate information free from bias is vital for effective CT (West et al., 2008). Therefore, we predict that higher scores on self-reported measures of both observing and non-reactivity will predict higher CT performance.
4. Fourth, it is hypothesized that self-reported observing or present-moment awareness will be positively related to the ability to (a) update the contents of working memory, (b) shift between different working memory representations, and (c) inhibit prepotent responses. Notably, previous research has linked greater skills of observation with better inhibitory control performance (Schmertz et al., 2009) and with better performance on tasks requiring participants to switch flexibly from one thought to another (Chambers et al., 2007). Higher self-reported observation skills has also been found to predict better performance on measures of working memory updating (Anicha et al., 2011).
5. Fifth, it is expected that non-reactivity will be positively related to inhibition and shifting. Non-reactivity has been associated with both behavioral and electrophysiological indicators of inhibition (Teper and Inzlicht, 2013) and greater cognitive flexibility (Anicha et al., 2011).
6. Sixth, it is expected that updating, shifting, and inhibition will be positively related to CT. CT may be most effective when information held in working memory is efficiently updated as new information is presented, when switching from one perspective to another is efficient, and when heuristic and biased responses and distractions are inhibited (West et al., 2008; Sanz de Acedo Lizarraga et al., 2012).
7. Finally, it is expected that the relationship between mindfulness and CT will be mediated by EF. To date no research has investigated the mechanisms by which mindfulness facilitates higher-order thinking skills. In light of theoretical work in the dual-processing tradition and research on mindfulness, self-regulation and higher-order thinking skills, we propose EF as a key mechanism driving this relationship. Establishing a mediational relationship between mindfulness, EF, and CT is necessary in order to support this proposition that the application of EF is a mechanism through which mindfulness facilitates CT performance (Baron and Kenny, 1986; Ostafin et al., 2013b).

## Materials and Methods

### Participants

One hundred and seventy eight university undergraduate psychology students (Mean age = 21.04; *SD* = 5.77; 39 males; 139 females) attending the National University of Ireland, Galway, participated in the study. *A priori* calculations using Soper’s (2015) SEM sample size calculator suggested a minimum sample size of 156 was required for analysis to yield adequate power. This was based on a small-medium anticipated effect size (0.2), six latent predictor variables, 20 observed variables and a desired power level of 0.8 at a probability level of 0.05. Participants were recruited by e-mail and an online participant recruitment system. They were awarded credit as part of their course requirement. Participant were over 18 and were required to have English as their first language or university level English (i.e., equivalent to 80 on TOEFL or 6.5 on IELTS; both are recognized tests of English as a foreign language). Exclusion criteria included those with alcohol and drug dependency (including prescribed sedation medications) and those with visual and hearing impairments not corrected to normal (as required for the computer tasks). This study was given ethical approval by the NUIG Research Ethics Committee.

### Study Design

The study employed a cross-sectional, individual differences design to examine the relationships between the observing and non-reactivity components of mindfulness, the updating, shifting, and inhibition components of EF and CT skills. SEM was used to test the model of specified relations between mindfulness, EF, and CT. SEM allows for multiple hypotheses to be tested simultaneously. SEM is best suited to test hypotheses patterns of direct and indirect relationships between theoretical concepts which contain observed and latent variables (MacCallum and Austin, 2000). In assessing EF, a latent variable approach (LVA) is best as it resolves the task impurity problem. This is a problem inherent in using individual EF tasks where non-executive processes contaminate the examination of executive processes (Miyake et al., 2000; Miyake and Friedman, 2012). LVA allows identification of how performance on multiple exemplar tasks is statistically shared (in this case the executive process), thus giving a truer measure of the construct underlying these tasks which can then be related to a target manifest variable, in this case CT (Del Missier et al., 2010). This means that two tasks for each of updating, switching, and inhibition were used in this study in order to compute latent variables for each EF. This approach has been used to investigate the role of in EF in decision-making (Del Missier et al., 2010), fluid intelligence (Friedman et al., 2006), and attentional difficulties (Friedman et al., 2007).

### Apparatus and Materials

All computer tasks were conducted on a Dell computer with a 15 in LCD monitor. The EF tasks were administered using the MATLAB software program which recorded reaction times and error commission.

#### Five Facet Mindfulness Questionnaire Short Form (FFMQ-SF; Bohlmeijer et al., 2011)

The Five Facet Mindfulness Questionnaire Short Form (FFMQ-SF) is a 24 items measure consisting of five subscales (observing, describing, acting with awareness, non-judging of inner experience, and non-reactivity to inner experience). The FFQM-SF employs a five-point Likert scale (e.g., 1 = never or very rarely true; 5 = very often or always true). For the purpose of the current study, the scores obtained for the observing and non-reactivity facets were the main focus. This multi-facet scale includes four observing items (e.g., “I pay attention to sounds, such as clocks ticking, birds chirping, or cars passing”) and five non-reactivity items (e.g., “I perceive my feelings and emotions without having to react to them”). The current study used non-reactivity and observing sub-scales to measure mindfulness, as these are the most widely agreed upon components of mindfulness analyzed in the empirical literature (Anicha et al., 2011). The non-reactivity sub-scale showed good reliability (α = 0.72) as did the observing sub-scale (α = 0.71).

#### Executive Functions Measures

Six executive tasks were administered to all participants to assess the three core components of EFs; *Shifting, Updating*, and *Inhibition*. For all tasks, participants were asked to respond as quickly and accurately as possible. The dependent variables for each task were the proportions of accurate responses. We followed Miyake et al. (2000) in using these dependent measures for all but the switching tasks. While switch costs are often examined in terms of reaction time, it is not uncommon to examine accuracy costs in terms of the proportion of accurate responses on switch trials (Sy et al., 2013). This method was chosen to avoid any potentially unique variance that could be attributed to reaction time and in light of evidence that reaction time switch costs do not reflect executive processes (Logan and Bundesen, 2003).

The following two tasks were used to measure *Shifting:*

##### Plus–minus task

The plus–minus task (Jersild, 1927; Spector and Biederman, 1976 adapted by Miyake et al., 2000) consists of three blocks in which 30 two-digit numbers are presented. In the first block participants are required to add 3 to each number. In the second block they are required to subtract 3 from each number. In the final block participant are required to alternate between adding 3 and subtracting 3 from each number (i.e., add 3 from the first number, subtract 3 from the next number and so forth). The proportion of accurate responses on switch trials was the dependent variable.

##### Number–letter task

The number–letter task (Miyake et al., 2000) is presented in three blocks. In the first block (32 trials) number–letter pairs are presented in the top two quadrants of a square grid on a computer screen. The participant is instructed to indicate whether the number is odd or even. In the second block (32 trials) the number–letter pair is presented in the bottom quadrants of the grid. In this block the participants are instructed to indicate whether a letter is vowel or consonant. In the final block number–letter pairs are presented in all quadrants of the grid. When they appear in the top quadrants, participants have to indicate whether the number is odd or even, and when they appeared in the bottom quadrants they had to indicate if a letter is a vowel or a consonant. Therefore in the third block, half of the trails require the participant to shift between the two types of categorisation. The dependent measure was the proportion of accurate responses on switch trials.

The following two tasks were used to measure *Updating*:

##### Tone monitoring task

In the tone monitoring task (Miyake et al., 2000) participants are presented with 25 tones of low, middle, and high frequency which are delivered over four trial blocks. The tones are presented for 500 ms each with an interval of 2500 ms. Each trial block consist of eight high-pitched tones (880 Hz), eight medium- pitched tones (440 Hz) and eight low- pitched tones (220 Hz), with the addition of 1 tone selected randomly from the other three for a total of 25. There were four blocks and six potential correct answers per block. Whenever one of the tones was played for the fourth time, the participant had to respond by pressing the corresponding key – 1 for low, 2 for medium, and 3 for high – while maintaining a count of the occurrences of the other tones in working memory. In order to avoid the impact of momentary mental lapse on task performance the tone count for each pitch automatically resets to 0 after incorrect responses. A different error tone would be heard on this occurrence. The dependent measure was the proportion of correct responses.

##### Letter-memory task

The letter-memory task (Miyake et al., 2000) involves constantly updating a series of letters in working memory. A series of letters are presented in succession in each trial for the duration of 2000 ms per letter. Participants are required rehearse the last four letters presented in the list, and then report the last four letters in the series at the end of each trial. The number of letters presented (5, 7, 9, or 11) varied randomly across trials to avoid habituation to trial length. The dependent measure is the proportion of correctly recalled letters.

The following two tasks were used to measure *Inhibition*:

##### Anti-saccade task

The anti-saccade task (Kane et al., 2001) requires participants to detect a sudden-onset visual cue and use that cue to direct their attention to a specific location that will contain a target stimulus. In the first block the cue appears in the same location as the target. In the second block the cue appears on the opposite side to the target. In this block when the cue predictably signals a location that does not contain the target, participants must voluntarily redirect their gaze from the cue to the target, ignoring the salient cue, and respond to the target. Participant must therefore maintain their goal (i.e., response to target) despite interference. The dependent measure was the proportion of correctly identified targets.

##### Stop-signal task

The stop-signal task (Miyake et al., 2000) requires inhibition of a learned response. This task is presented in two blocks. In the first block participants are presented with 24 words one-by-one (e.g., duck, gun), they are instructed to indicate if the word was an animal or not by means of button presses (i.e., left = animal, right = not). In the second block participants were instructed not to respond when they heard a tone. If no tone was played they were required to respond as they did in the first block. In all trials a fixation point appears on the screen 500 ms prior to the presentation of the stimulus and participants are given up to 1500 ms to make their response. The dependent measure was the proportion of correctly withheld responses for the stop trials.

##### Halpern Critical Thinking Assessment (Halpern, 2010)

The Halpern Critical Thinking Assessment (HCTA) assesses thinking in everyday, easy to relate to scenarios. It taps both the motivational and behavioral parts of CT by including both open-ended and multiple choice questions (Ku, 2009). The assessment consists of 25 everyday situations that the participant must analyze and critique. Following these situations are 25 open-ended questions followed by 25 specific questions that assess the reasoning behind each answer. Across these questions five sub-categories of thinking skills must be applied including argument analysis skills, verbal reasoning skills, hypothesis testing skills, likelihood and uncertainty judgment skills and decision making/problem solving skills. There are five items for each sub-category of CT with the maximum points possible for each sub-category varying. Scoring of the HCTA was carried out by three trained graders. The scoring guide provides answers for forced-choice questions while open-ended questions are graded according to specific grading prompts (for more detail see Dwyer et al., 2012). Greater scores are awarded to more accurate and comprehensive answers and total scores can range from 0 to 194 (Halpern, 2010). The internal reliability of the HTCA was found to be adequate with a Cronbach’s alpha coefficient of 0.79 in this study which corresponds to robust reliability found in other samples (Halpern, 2010; Dwyer et al., 2012).

### Procedure

All participants first completed the HCTA and the FFMQ-SF questionnaire online (along with other measures not pertinent to this study) at a separate location (e.g., home or campus computers). The online questionnaires were administered using Survey Gizmo. At a later date, within a month of online testing, all participants were administered six computerized EF tasks in a laboratory cubicle at the School of Psychology building at NUI Galway. Here, participant consent was again obtained (following provision of informed consent online). A pseudo-random counterbalancing strategy was applied to the task order, involving six orders with no two orders repeating the order of consecutive pairs of tasks. This would have kept any EF depletion or fatigue effects constant across tasks and participants. At the end of each task participants were prompted to call the researcher, the researcher then applied the specified order when initiating the next task. The laboratory session took approximately 1 h to complete for each participant.

### Analytic Approach

The approach to analysis consisted of four stages. First, a series of measurement models were used to evaluate the mindfulness and EF constructs. Second, a structural model of EF and CT was evaluated. Third, a structural model of dispositional mindfulness, EF, and CT was evaluated based on the structural model established in the second stage. Finally, a multiple mediator analysis was carried out by examining the direct and indirect effects between the mindfulness factors of Observing and Non-reactivity, the EF factors of Updating, Inhibition and Shifting and CT in order to determine the nature of any mediation/non-mediation present.

## Results

A summary of the descriptive statistics for variables representing mindfulness, EF, and CT performance can be seen in **Table 1** along with correlations between them. Most variables had relatively low values for skewness and kurtosis except for the Number–Letter, Letter Memory, and Anti-saccade tasks. Arc Sin transformations, which are often employed with proportional measures, were successful in achieving acceptable kurtosis and skewness values for the EF task dependent measures. Due to computer program malfunctions, some participants (*N* = 7) did not have scores for specific EF tasks. EM substitution was carried out in order to treat missing data since each of these participants only had missing scores for one particular task. Reliability for all measures was assessed using Cronbach’s alpha and was adequate for all except the Stop Signal task. Correlations among the variables were consistent with previous studies (Miyake et al., 2000; Friedman et al., 2006, 2008; Del Missier et al., 2010). Descriptive statistics, correlation analyses, reliability analyses, and EM substitution were all carried out using SPSS 20 (IBM corp, 2011). Measurement models, structural models and mediation analyses were conducted in AMOS 22 (Arbuckle, 2013).

**Table 1:** Descriptive statistics and correlations for mindfulness, executive functions, and critical thinking^1^.

	*M*	*SD*	*α^2^*	1	2	3	4	5	6	7	8	9
1. Non-reactivity	14.01	3.13	0.72	1								
2. Observing	13.35	2.96	0.71	0.09	1							
3. Tone monitoring	0.29	0.25	0.86	0.14	0.24^∗∗^	1						
4. Stop signal	0.76	0.17	0.55	0.00	0.12	0.25^∗∗^	1					
5. Plus–minus	0.67	0.32	0.96	0.02	0.06	0.35^∗∗^	0.09	1				
6. Number–letter	0.91	0.13	0.95	-0.08	0.07	0.28^∗∗^	0.24^∗∗^	0.20^∗∗^	1			
7. Letter memory	0.66	0.18	0.75	-0.04	-0.05	0.17^∗^	0.08	0.12	0.06	1		
8. Anti-saccade	0.86	0.13	0.91	-0.09	0.12	0.42^∗∗^	0.28^∗∗^	0.27^∗∗^	0.26^∗∗^	-0.05	1	
9. Critical thinking	107.53	16.51	0.79	-0.12	0.14	0.20^∗∗^	0.14	0.31^∗∗^	0.20^∗∗^	0.09	0.24^∗∗^	1

*^1^Mean and standard deviation for untransformed variables are presented while correlations presented are between transformed variables (^∗^p < 0.05; ^∗∗^p < 0.01).*

*^2^Cronbach’s alpha.*

### Measurement Models

#### Mindfulness

The two factor model of the FFMQ-SF using sub-scales of non-reactivity and observing indicated good model fit. The chi-square test was non-significant (33.12, *p* = 0.16) with TLI (0.97), IFI (0.98) and CFI (0.98) values all above 0.95 and a RMSEA value of 0.04 [0.00 -0.08]. Furthermore, all factor loadings ranged between 0.46 and 0.85. Notably, it was a much better fit than a one factor model for which the chi-square test was significant (200.161, *p* < 0.001), the TLI (0.22), IFI (0.43) and CFI (0.42) values were all below 0.45 and the RMSEA value was above 0.08 (0.19 [0.00 -0.08]).

#### Executive Function

Confirmatory factor analysis (CFA) was used to compare models with one, two, three or no related factors. This was done by first testing the full three-factor model and then testing the alternative models nested within the full model, achieved by fixing specific correlations among the latent variables in the following ways – for the one factor models, all correlations were fixed to 1.0, for the two factor models, the correlation between the various pairs of unitary latent variables was fixed to 1.0 while the other two were allowed to vary freely and for the model with no relationships among the three factors (i.e., independence), all correlations were fixed to zero. It was expected that the full three factor model would provide the best fit to the data. Of these possibilities, a nested two factor model with the covariance between the Shifting and Updating factors constrained to one provided the most adequate fit to the data. However, given the low factor loadings of the Plus–Minus and Number–Letter tasks on the Shifting factor, it was decided to drop the Shifting factor from further analyses. The resulting model demonstrated adequate fit with a non-significant chi-square test (3.83, *p* = 0.05), and IFI and CFI values of 0.95 (although the TLI value was an inadequate 0.67). This model had an AIC value of 21.83 and AIC values for the competing models were all worse, ranging from 36.20 for the nested two factor model described above to 124.23 for the three independent factors model.

### Structural Models

#### Executive Function and Critical Thinking

Following Miyake et al. (2000), this stage involved adding CT performance as a manifest variable to the EF model supported in the previous stage. Potential paths from the latent variables to the CT manifest variable were evaluated. Models with either two or one path(s) (i.e., paths from both inhibition and updating, or paths from either inhibition or updating) to the CT manifest variable were compared to determine the paths necessary to provide the best fit to the observed data. The two-path model fit the observed data best (see **Table 2**) and demonstrated a strong significant relationship between Updating and CT (β = 3.25, *p* = 0.04). In order for this model and the one-path model connecting Updating and CT to identify, the error variance of CT was fixed to 0.001. This is acceptable in cases where a model will not identify due to negative error variance but the estimate of negative error variance is not statistically significantly different from 0 (Dillon et al., 1987).

**Table 2:** Fit indices for structural models of executive functions and critical thinking.

Model	*df*	χ^2^	*p*	IFI	CFI	TLI	RMSEA	AIC
Two path model	4	4.98	0.29	0.99	0.99	0.97	0.04 [0.00 -0.13]	26.98
One Path – Inhibition	4	7.59	0.11	0.95	0.95	0.87	0.07 [0.00 -0.15]	29.59
One Path – Updating	5	41.02	0.00	0.52	0.49	-0.02	0.20 [0.15 -0.26]	61.02

#### Mindfulness, Executive Function, and Critical Thinking

This stage involved adding the Observing and Non-reactivity facets of dispositional mindfulness as manifest variables to the model supported in the previous stage. A model with the hypothesized paths was compared against two reference structural models with one assuming no relation between both mindfulness facets and the three latent EF variables and one assuming that each facet is related to each EF. For the hypothesized model to be supported, it needed to demonstrate a better fit to the data than the model assuming no relations and the fit had to be no worse than the full path model. This analysis strategy is similar to that employed by Del Missier et al. (2010). To achieve the best possible fit, the error variances for the Tone Monitoring scores and the Letter Memory scores respectively were allowed to co-vary with the error variance for the Anti-saccade task, as suggested by the modification indices. Examination of the model fit indices demonstrated that the hypothesized model was supported (see **Table 3**). Parameter estimates revealed a significant positive relationship between Observing and Inhibition and a weak but significant negative relationship between Non-reactivity and CT (see **Table 4**).

**Table 3:** Fit indices for structural models of mindfulness, executive functions, and critical thinking.

Model	*df*	χ^2^	*p*	IFI	CFI	TLI	RMSEA	AIC
Hypothesized	8	13.62	0.09	0.94	0.93	0.82	0.06 [0.00 -0.12]	53.62
No paths	12	31.58	0.002	0.79	0.76	0.59	0.10 [0.06 -0.14]	63.58
Full path	7	13.52	0.06	0.93	0.92	0.76	0.07 [0.00 -0.13]	55.52

**Table 4:** Estimates in the multiple mediation model.

Type of effect	*b*	*SE*	β	*BC 95% CI*	*p*
**Direct effects**					
Observing → Updating	0.44	0.53	0.04	[-0.33, 1.81]	0.26
Observing → Inhibition^∗∗^	1.77	0.57	0.23	[0.65, 2.85]	0.001
Observing → Critical Thinking	-0.06	1.17	-0.01	[-1.85, 1.30]	0.92
Non-reactivity → Inhibition	0.87	0.53	0.12	[-0.11, 2.01]	0.08
Non-reactivity → Critical Thinking^∗^	-0.91	0.58	-0.17	[-2.13, -0.07]	0.04
Updating → Critical Thinking^∗∗∗^	0.82	0.61	1.47	[0.37, 2.40]	0.000
Inhibition → Critical Thinking^∗∗^	0.31	0.60	0.43	[0.03, 1.69]	0.009
**Specific indirect effects**					
Non-reactivity → Inhibition → Critical Thinking^∗^	0.27	0.46	0.05	[0.01, 2.35]	0.04
Observing → Inhibition → Critical Thinking^∗∗^	0.55	0.99	0.10	[0.04, 2.97]	0.009
Observing → Updating → Critical Thinking	0.36	0.57	0.07	[-0.31, 1.48]	0.25
**Total indirect effects**					
Non-reactivity → Inhibition → Critical Thinking^∗^	0.27	0.46	0.05	[0.01, 2.35]	0.04
Observing → Inhibition + Updating → Critical Thinking	0.91	1.14	0.16	[-0.22, 2.82]	0.12
**Total effects**					
Non-reactivity → Inhibition → Critical Thinking	-0.64	0.38	-0.12	[-1.40, 0.10]	0.09
Non-reactivity → Critical Thinking					
Observing → Inhibition + Updating → Critical Thinking	0.85	0.42	0.15	[0.05, 1.69]	0.04
Observing → Critical Thinking^∗^					

*^∗^p < 0.05; ^∗∗^p < 0.01; ^∗∗∗^p < 0.001.*

### Multiple Mediation Model

**FIGURE 1:**
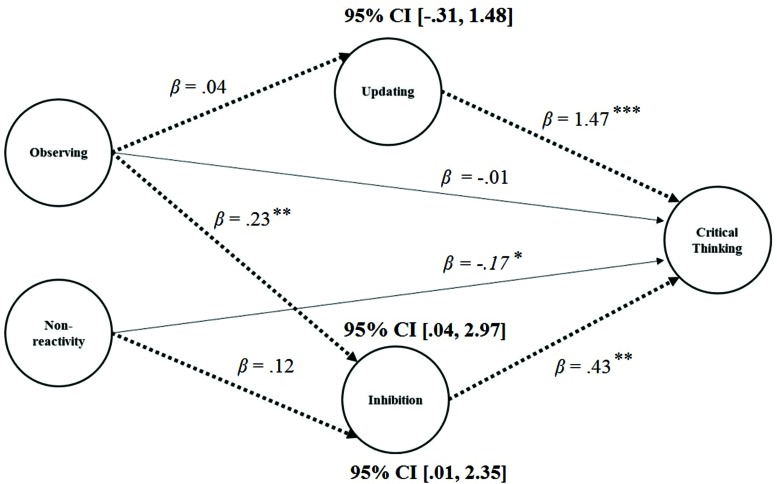
**Multiple mediation model of mindfulness facets, executive functions, and critical thinking with standardized direct effects and 95% confidence intervals (CIs), using bias corrected bootstrapping for indirect effects**. ^∗^*p* < 0.05; ^∗∗^*p* < 0.01; ^∗∗∗^*p* < 0.001.

In order to investigate whether the effects of the Observing and Non-reactivity facets of dispositional mindfulness on CT were mediated by EF, a multiple mediation analysis (MacKinnon et al., 2007) was carried out. **Figure 1** displays the model which was examined. The first step in the multiple mediation analysis involved quantifying the *direct effects* of mindfulness on both EF and CT respectively. More specifically, it required examining the paths leading directly from the dispositional mindfulness facets of Observing and Non-reactivity to the EF latent variables, and from both the mindfulness and EF factors to CT. Then the *specific indirect* effects were quantified. Here these are defined as the effects leading from a mindfulness factor via each EF factor to CT. Following this, the *total indirect effects* were calculated by summing the specific indirect effects. Lastly, the direct effect of each mindfulness factor (i.e., Observing and Non-reactivity respectively) on CT and their corresponding specific indirect effect were summed to find the *total effects*. These effects were estimated, along with their 95% confidence intervals (CIs), using bias corrected bootstrapping with 5,000 draws specified, as recommended (see **Table 4**; Kline, 2011).

The different patterns of mediation/non-mediation present can be described using an approach developed by Zhao et al. (2010). The significance of both the indirect and direct effects were examined to determine whether meditation is present and, if it was, whether it was complementary (where both indirect and direct effects are significant and the multiplication of their estimates is positive), competitive (where both indirect and direct effects are significant and the multiplication of their estimates is negative) or indirect only [where there is no significant direct effect but the indirect effects is significant – full mediation in Baron and Kenny’s (1986) terms]. It was found that for the path leading from Non-Reactivity to CT through Inhibition that there was evidence for competitive mediation since the direct effect of Non-Reactivity on CT was significant but negative while its specific indirect effect through Inhibition was significant and positive. For the path from Observing to CT through Inhibition, evidence for indirect-only mediation was present as this specific indirect path was significant and positive but the direct path from Observing to CT was not significant. Since the indirect path from Observing to CT through Updating was not significant either, Updating cannot be considered a mediator of the relationship between Observing and CT in this model. Overall, the model explained 36.3% of the variance in CT. There was no difference in strength between the two significant specific indirect effects observed which indicates that they both affect CT performance to a similar extent (*p* = 0.22).

## Discussion

This study sought to investigate the claim that higher dispositional mindfulness facilitates CT performance. This claim was based on previous research on the cognitive benefits of mindfulness and the self-regulation of higher-order cognition more generally and insights from the Default Interventionist theory of Hisgher-order Cognition. Furthermore, this study aimed to examine whether EF performance operates as a mechanism through which mindfulness facilitates CT. Relations between the observing and non-reactivity facets of mindfulness, the EF processes of updating, shifting and inhibition, and CT performance were examined using SEM. This analysis revealed a number of interesting findings in relation to the specified hypotheses.

Our first hypothesis stated that the two-component model of mindfulness would be found to be a better fit than a one-factor solution. This conceptualisation made use of the observing and non-reactivity subscales of the Five Factor Mindfulness Questionnaire. These sub-scales represent the most supported operational definition of mindfulness and have been found to have dissociable cognitive correlates (Anicha et al., 2011). Consistent with our first hypothesis, the two-component model of mindfulness was supported and used in the subsequent SEMs.

Our second hypothesis concerned the structure of EF. Specifically, consistent with Miyake et al. (2000) unity and diversity model of EF and subsequent research supporting this (Del Missier et al., 2010; Miyake and Friedman, 2012), it was hypothesized that the measurement model for EF would support a three-factor model structure which proposes that updating, inhibition, and shifting are related but distinct components of EF. EF was assessed using a battery of tasks measuring the skills of updating, inhibition and switching to which a latent variable analysis was applied. A two-factor solution was found to be optimal in the current study, with the updating and inhibition factors retained as the inclusion of the shifting tasks led to problems in identifying a positive definite covariance matrix. This could be due to the ceiling effects found for these tasks in the current sample. Factor loadings were in the range reported in previous studies (Miyake et al., 2000; Del Missier et al., 2010).

The third hypothesis focused on the direct effects of mindfulness on CT. It was expected that both observing and non-reactivity would be positively related to CT. Results indicated no direct effect of observing on CT. However, there was a positive, indirect effect of observing on CT that was mediated by inhibition, suggesting that observing influences EF which in turn influences CT ability. This indirect effect is discussed in more detail below in regard to our final hypothesis. Converse to our hypotheses, non-reactivity was found to be significantly negatively related to CT. This is an interesting finding which suggests the possible existence of mediators not accounted for by the model which may have a debilitative effect on CT performance. Potential candidates include worry and repetitive thought, emotional regulation and positive mood, and non-elaborative processing. For example, mindfulness has been shown to reduce habitual worry and evidence suggests that a key mechanism underlying this is the negative relationship between non-reactivity and repetitive thought (Evans and Segerstrom, 2011). Furthermore, responsibility to continue thinking, a process underlying habitual worry, has been shown to be important for effective CT (Sugiura, 2013). It is possible that in reducing repetitive thought and worry, non-reactivity could indirectly impair CT performance. Similarly, as regards the possible role of emotional regulation, non-reactivity is thought to require executive control to inhibit the elaboration of acute affective cues (Teper et al., 2013), and this has been shown to result in the down-regulation of negative affect and increases in positive affect (Chambers et al., 2009). However, this pattern of emotional regulation may result in sub-optimal conditions for CT performance since negative affect tends to facilitate the engagement of EF and positive affect increases the probability of intuitive thinking (Bolte et al., 2003; Fiedler et al., 2003; Teper et al., 2013; Inzlicht et al., 2015). It is also possible that the measure of non-reactivity used in the current study captures a tendency for non-elaborative processing beyond just affective cues. The questionnaire items used to assess non-reactivity focus on the ability to “let go” soon after the experience of distressing thoughts, rather than persisting with the thoughts. This dispositional tendency may lead to less cognitive effort being engaged when thinking in stressful, problem situations, which a test such as the Halpern Critical Thinking Assessment could be considered to be. Cognitive effort is vital for effective CT performance (Stanovich, 2011) and it is possible that high levels of non-reactivity are associated with low levels of cognitive effort in some situations. It is possible that any one of these factors, or a combination, could account for the negative relationship found between non-reactivity and CT in the current study. However, further research is needed to replicate the findings observed in the current study and examine these and other explanatory mechanisms in more detail.

Our fourth hypothesis specified direct relations between observing and the EF components. It was expected that observing would be positively related to updating, inhibition, and shifting. Observing was not found to predict updating in the current study, and it was not correlated with shifting performance, but observing did predict inhibition as hypothesized. Much previous research has linked mindfulness skill with enhanced inhibition (Heeren et al., 2009; Sahdra et al., 2011; Greenberg et al., 2012b). Predictions of a link between observing and inhibition were based on process descriptions of mindfulness, which emphasize the inhibition of elaborative processing in order to keep one’s attention focused on observing the present-moment (Bishop et al., 2004). This is an important finding as the proposed relationship between mindfulness and inhibition is often invoked in explaining how mindfulness disrupts automatic thinking across studies of the outcomes of mindfulness (e.g., Frewen et al., 2007; Kang et al., 2012; Ostafin and Kassman, 2012; Ostafin et al., 2013a). Studies have also suggested the benefits of mindfulness practice for working memory operation and the link between observing and working memory updating has been theorized previously (Jha et al., 2010; Anicha et al., 2011; van Vugt and Jha, 2011; Mrazek et al., 2013). The lack of a direct relationship between observing and updating in the current study may reflect the approach the study adopted. Most studies focusing on the effects of mindfulness on working memory have involved training interventions and the only finding of a positive relationship between observing and working memory updating from a dispositional perspective may have been due to perceptual rather than executive benefits (Anicha et al., 2011). It is possible that specific mindfulness training is required in order for individual differences in working memory updating to emerge in relation to skills in observing.

The fifth hypothesis, concerning the relationship between non-reactivity and inhibition was not supported, contrary to previous findings from mindfulness training studies (Anicha et al., 2011; Teper et al., 2013). Regarding the relationship between non-reactivity and shifting, no significant relationships were observed. It is surprising to find no direct effects of non-reactivity on any component of EF since the engagement of executive control to suppress the elaboration of affective cues is considered to be central to non-reactive information processing (Teper and Inzlicht, 2013). However, a recent study found a significant but small negative effect of non-reactivity on self-reported behavior regulation, which includes items related to inhibition and shifting (Short et al., 2015). Non-reactivity skills have been shown to take longer to develop than observing skills in training studies (Lilja et al., 2012) and perhaps more explicit guidance and practice are needed to demonstrate significant links between non-reactivity and EF. Furthermore, the self-report of non-reactivity outside of mindfulness training contexts may be different to the self-report of this ability after some exposure to mindfulness practices, and thus relations between mindfulness dispositions and EF may differ across different study contexts (Van Dam et al., 2009).

Our sixth hypothesis stated that the EF components would each exert significant direct, positive effects on CT. Consistent with our hypothesis, both updating and inhibition were significantly and positively associated with CT in the final SEM model. The effect of updating on CT was stronger than that of inhibition. One possible explanation for this pattern of findings is that updating is more important for sustained high level performance on the Halpern task. For instance, once the initial intuitive response to a situation in the Halpern Critical Thinking Assessment is inhibited, updating is continuously engaged as the contents of working memory are manipulated and revised and as more information is gleaned from the question. Another possibility is that the low reliability for the Stop Signal task contributed to the weaker effect of inhibition. Importantly, this is the first study to demonstrate a relationship between specific EFs and CT performance. This finding aligns with previous research suggesting the importance of working memory for effective CT (Dwyer et al., 2014) and is in line with the Default Interventionist theory which emphasizes the necessity of working memory operations for Type 2 processes such as CT (Evans and Stanovich, 2013).

Finally, it was hypothesized that the relationship between mindfulness and CT would be mediated by EF. Using SEM to conduct a multiple mediation analysis, it was found that there was evidence for inhibition mediating the relationships between CT and both observing and non-reactivity. The relationship between observing, inhibition and CT is considered to be indirect-only mediation [or full mediation in Baron and Kenny’s (1986) terms] since no direct effect of observing on CT was found. This finding suggests that the entirety of the effect of present-moment mindful observation on CT is due to it being positively related to inhibition. Evidence for competitive mediation was found for the relationship between non-reactivity, inhibition, and mindfulness. This means that while there was a negative direct effect of non-reactivity on CT, there was a positive indirect effect of non-reactivity on CT that was mediated by inhibition. This positive indirect effect makes sense considering the fact that non-reactivity has been assumed to reduce automatic responding and promote more reflective responses (Peters et al., 2015). This competitive relationship suggests the presence of additional mediators not accounted for by the model, as discussed above, which act independently of the positive indirect relationship (Zhao et al., 2010). The observation that inhibition acts as a mediator of both components of mindfulness in this model provides evidence for inhibition as a core mechanism of the effect of mindfulness on CT. This mechanism has been suggested in several previous studies showing facilitative effects of mindfulness on higher-order cognition (Cottone and Javier, 2007; Ruedy and Schweitzer, 2011; Ostafin et al., 2013b; Wen et al., 2013) but has never been explicitly demonstrated before. This finding ties the relationship between mindfulness, EF, and CT closely to the Default Interventionist Dual Process Theory of Higher-order Cognition which posits that it is through the inhibition of the prepotent tendency to accept the outcomes of Type 1 processes that Type 2 higher-order cognitive processes such as CT are engaged (Stanovich and Toplak, 2012).

This study had several strengths. Methodologically speaking, it employed objective behavioral measures of both EF and CT. It had a relatively large sample size to allow for the reliable identification of effects using SEM (Soper, 2015). The use of SEM also accounted for the task impurity problem in the measurement of EF and allowed multiple mediation to be examined. While previous research has suggested that mindfulness can facilitate higher-order thinking skills in general, its effect on CT specifically had not been examined and no attempts had been made to identify the mechanisms driving this relationship. This study also sheds light on the extent to which particular executive processes are implicated in CT. By identifying links between literature on mindfulness and self-regulation, and studies on the self-regulation of higher-order cognition it was hypothesized that EF would mediate the effect of mindfulness on CT. Consistent with Evans and Stanovich’s (2013) default interventionist dual process theory of higher-order cognition, it appears that mindfulness may be enabling the engagement of Type-2 processing as a result of its positive effects on EF, particularly inhibition. This study adds to the very few studies that have focused on the effects of dispositional mindfulness in psychologically healthy individuals (Petrocchi and Ottaviani, 2015) and increases our knowledge regarding the mechanisms by which mindfulness enhances higher-order cognition.

However, there are some weaknesses to this study. As with any cross-sectional study, caution must be taken in interpreting these findings as causal. However, as the model fit was good, the hypothesized causal relations can be said to be plausible (Bollen and Pearl, 2013) and can be used as the basis for further experimental work that examines causal hypotheses in controlled settings. The use of a self-report measure to assess dispositional mindfulness and the use of non-practitioners of mindfulness mean that further research is needed before the results of the current study can be generalized to the practice of mindfulness. Furthermore, debate continues regarding the validity of self-report measures of dispositional mindfulness. While investigations involving the FFMQ suggest that the non-reactivity facet represents its corresponding aspect of mindfulness well, divergent views exist regarding how best to measure the present-moment attention aspect with some studies supporting the acting with awareness facet (Rau and Williams, 2015) and others supporting the observing facet (Anicha et al., 2011). It is clear that further refinement of the FFMQ is required to increase its validity (Petrocchi and Ottaviani, 2015). As regards the measurement of EF, despite adequate piloting a number of participants did not understand the switching task, while others found it too easy which produced a ceiling effect. This led to low factor loadings on the shifting factor and much better fit when this factor was dropped, thus not supporting the common conceptualisation of the unity/diversity model of EF. Any Speed-Accuracy Trade-Off advantage, sometimes observed in more mindful individuals (van Vugt and Jha, 2011), would not have been captured by the proportional accuracy dependent variables employed here. However, this has only been observed in individuals trained in mindfulness and thus may not have had an effect in the current study. Finally, the measures of EF we employed were purely performance based. It has been suggested that including both self-reported measures of EF and self-regulation and performance-based measures is important for increasing ecological validity (Toplak et al., 2013). Future investigations could include measures such as the Behavior Rating Inventory of Executive Functions which has been shown recently to mediate the relationship between mindfulness and both positive and negative affect independently of and to a greater extent than performance-based EF tasks (Short et al., 2015).

In summary, dispositional mindfulness appears to facilitate, albeit weakly, CT performance and this effect is mediated by the inhibition component of EF. However, this relationship is complex as the non-reactivity facet of mindfulness appears to have a competing negative effect on CT through as yet unidentified mediators. These findings suggest many possibilities for future research. In order to support the claim that mindfulness can improve CT in educational settings (Shapiro et al., 2011), careful intervention research will be needed. Applying experimental designs to this research in both short-term lab settings and longer-term intervention contexts will be useful in learning more about the developmental trajectory of the relationship between mindfulness and CT and in identifying relevant mediators other than EF. Further focus on the role of EFs is important but additional mediators are likely and must be investigated. It would certainly be interesting to compare current CT instructional courses with similar courses infused with mindfulness lessons (e.g., Bush, 2011). However, it is important to first continue investigations of the basic relationships between mindfulness and higher-order cognitive skills in typically developing individuals and the mechanisms underlying any significant relationships found.

## Author Contributions

Dr. MH and CN designed the study. CN collected the data, carried out data analysis and interpretation of the results and wrote the manuscript. Dr. MH reviewed, revised and approved the content of the manuscript. Prof. BB reviewed, gave advice and approved the statistical analyses.

## Conflict of Interest Statement

The authors declare that the research was conducted in the absence of any commercial or financial relationships that could be construed as a potential conflict of interest.
